# Integrating extended reality into preoperative neurosurgical planning: exploratory evaluation of planning-related behavior in an instrumented hybrid framework

**DOI:** 10.3389/fdgth.2026.1978547

**Published:** 2026-10-28

**Authors:** Anna Drechslerová, Georg Widhalm, Gilbert Hangel, Niloofar Rajabi, Jan Huber, Günther Grabner, Wolfgang Bogner, Andreas Daniel Hartl, Barbara Kiesel

**Affiliations:** 1 Department of Engineering & IT, Carinthia University of Applied Sciences, Villach, Austria; 2 High-Field Magnetic Resonance Center (HFMRC), Department of Biomedical Imaging and Image-Guided Therapy, Medical University of Vienna, Vienna, Austria; 3 Department of Neurosurgery, Medical University of Vienna, Vienna, Austria; 4 Comprehensive Center for Artificial Intelligence in Medicine (CAIM), Medical University of Vienna, Vienna, Austria; 5 Christian Doppler Laboratory for MR Imaging Biomarkers (BIOMAK), Medical University of Vienna, Vienna, Austria

**Keywords:** extended reality, human–computer interaction, medical visualization, mixed reality, neurosurgical planning, planning-related behavior, quantitative assessment, surgical planning

## Abstract

**Background:**

Extended reality (XR) has increasingly been investigated for visualization and preoperative planning in neurosurgery. However, evaluation has often focused on visualization, usability, technical performance, and task outcomes, while comparatively less attention has been given to quantifying observable planning-related behavior.

**Methods:**

We developed and evaluated an instrumented hybrid desktop–XR framework integrating conventional image-based planning with immersive exploration of patient-specific neurosurgical anatomy. The framework synchronized selected visualization and interaction states between a 3D Slicer-based desktop environment and an XR workspace and captured spatial, anatomical, workflow, interaction, and subjective measures. Twenty participants with different levels of clinical experience evaluated two patient-specific tumor cases.

**Results:**

All participants completed both evaluation cases without session-ending technical interruption. Entry–tumor-center directions showed substantial convergence, with directional convergence of 0.881 for the GBM case and 0.965 for the LGG case, while anatomical structure selection showed fair overall agreement (Fleiss’ κ=0.397). Workflow, interaction, and subjective measures provided complementary information on task execution and user experience.

**Conclusion:**

The instrumented hybrid desktop–XR framework demonstrated technical and procedural feasibility under the study conditions for capturing complementary quantitative measures of spatial planning, anatomical selection, workflow, interaction, and subjective experience. These findings demonstrate that selected components of preoperative planning can be made observable and quantifiable within an XR-supported workflow. The measures should currently be regarded as descriptive behavioral measures rather than validated indicators of planning quality, expertise, or clinical correctness. The framework provides a methodological basis for subsequent validation using larger cohorts, more diverse cases, conventional-workflow comparisons, and expert or clinical reference criteria.

## 1 Introduction

Preoperative neurosurgical planning requires interpretation of patient-specific anatomy and consideration of possible surgical access routes within complex three-dimensional spatial relationships (1, 2). In neuro-oncological surgery, this includes consideration of lesion location, eloquent cortical and subcortical regions, functional mapping information, and anatomical constraints when evaluating potential surgical access routes (1, 3–6). The patient-specific anatomical configuration may constrain the set of feasible approaches, while surgical strategy must balance tumor access and extent of resection against the risk of neurological injury (3, 6, 7). Preoperative imaging characteristics have also been investigated in relation to surgical complexity and postoperative outcome (8).

Computer-assisted methods support this process through multimodal visualization, segmentation, image-guided navigation, and patient-specific modeling (1, 2, 9–12). Nevertheless, planning remains an iterative decision process in which surgeons inspect patient-specific anatomy, consider alternative access routes, and assess structures relevant to the proposed approach (1, 13). Conventional image-based environments provide detailed anatomical information, but complex three-dimensional relationships may require users to mentally integrate information across multiple two-dimensional views and reconstructed models.

Extended reality (XR) has increasingly been investigated as a complementary approach for neurosurgical visualization, education, planning, and navigation (9, 14–19). Immersive visualization can provide spatial perspectives that are difficult to obtain from conventional displays and may support inspection of tumor morphology, vascular anatomy, functional regions, and potential access directions (20–25). Previous studies have demonstrated mixed-reality cranial planning and case review, integrated presurgical mapping and mixed-reality navigation, and real-time technical integration between 3D Slicer and Microsoft HoloLens 2 (21, 22, 26). XR-supported preoperative planning has also been investigated in clinical and survey-based studies (27). However, evaluations of these systems have largely focused on usability, technical performance, visualization, timing, task performance, registration or navigation accuracy, and clinical or approach-related effects (16, 21, 26, 27). Comparatively less attention appears to have been given to systematically capturing how users interact with XR-supported planning environments and how planning-related actions and choices can be quantitatively characterized (16). This gap is particularly relevant to preoperative planning, where important spatial and anatomical decisions are made through iterative interaction with complex three-dimensional representations.

A further challenge is the integration of immersive visualization with established clinical planning workflows (22, 26). Neurosurgical planning relies on both detailed image-based interpretation and three-dimensional spatial reasoning (1, 4, 12). A hybrid desktop–XR approach may therefore complement rather than replace conventional planning by preserving established image-based tools while enabling immersive exploration of patient-specific anatomy. Such an environment also creates an opportunity to capture aspects of planning that are otherwise implicit. Although internal surgical reasoning cannot be observed directly in this setting, selected components of planning can be operationalized through observable actions. Previous work on surgical process modeling, inter-surgeon variability, and computer-assisted intervention has demonstrated the value of structured behavioral and workflow data for characterizing procedural variation and surgical activity (28–33).

Building on this perspective, we developed an instrumented hybrid desktop–XR framework that combines conventional desktop-based image review with immersive exploration of patient-specific anatomy. The framework synchronizes selected visualization and interaction states between a 3D Slicer-based planning environment and an XR workspace, while capturing complementary measures of spatial planning, anatomical selection and prioritization, workflow, interaction, and subjective assessment. These measures are intended to characterize observable planning-related behavior, rather than to directly measure surgical expertise, planning quality, or clinical correctness.

The objectives of this exploratory study were therefore to evaluate the technical and procedural feasibility of the synchronized desktop–XR framework and to investigate whether it can capture quantitative variation in observable planning-related behavior across participants with different levels of clinical experience. By making selected otherwise implicit components of preoperative planning observable and quantifiable, the framework provides a basis for subsequent clinically grounded validation of XR-supported neurosurgical planning.

## 2 Materials and methods

### 2.1 Synchronized desktop–XR planning framework

A synchronized desktop–XR planning framework was developed to combine conventional image-based review with immersive visualization of patient-specific anatomy within a shared planning workflow. The desktop component was implemented as a custom module in 3D Slicer 5.10.0 (34). The XR component was implemented in Unity 2022.3.36f1 (Unity Technologies, San Francisco, CA, USA) and rendered on Microsoft HoloLens 2 (35) using Holographic Remoting (36). A Python-based Transmission Control Protocol (TCP) server maintained communication between the two environments using structured JavaScript Object Notation (JSON) messages. Synchronized parameters included anatomical structure visibility, model transparency, slice-plane positions, and camera-rotation commands. A conceptual overview of the instrumented hybrid desktop–XR environment and the captured planning-related measures is provided in Figure 1.

**Figure 1:**
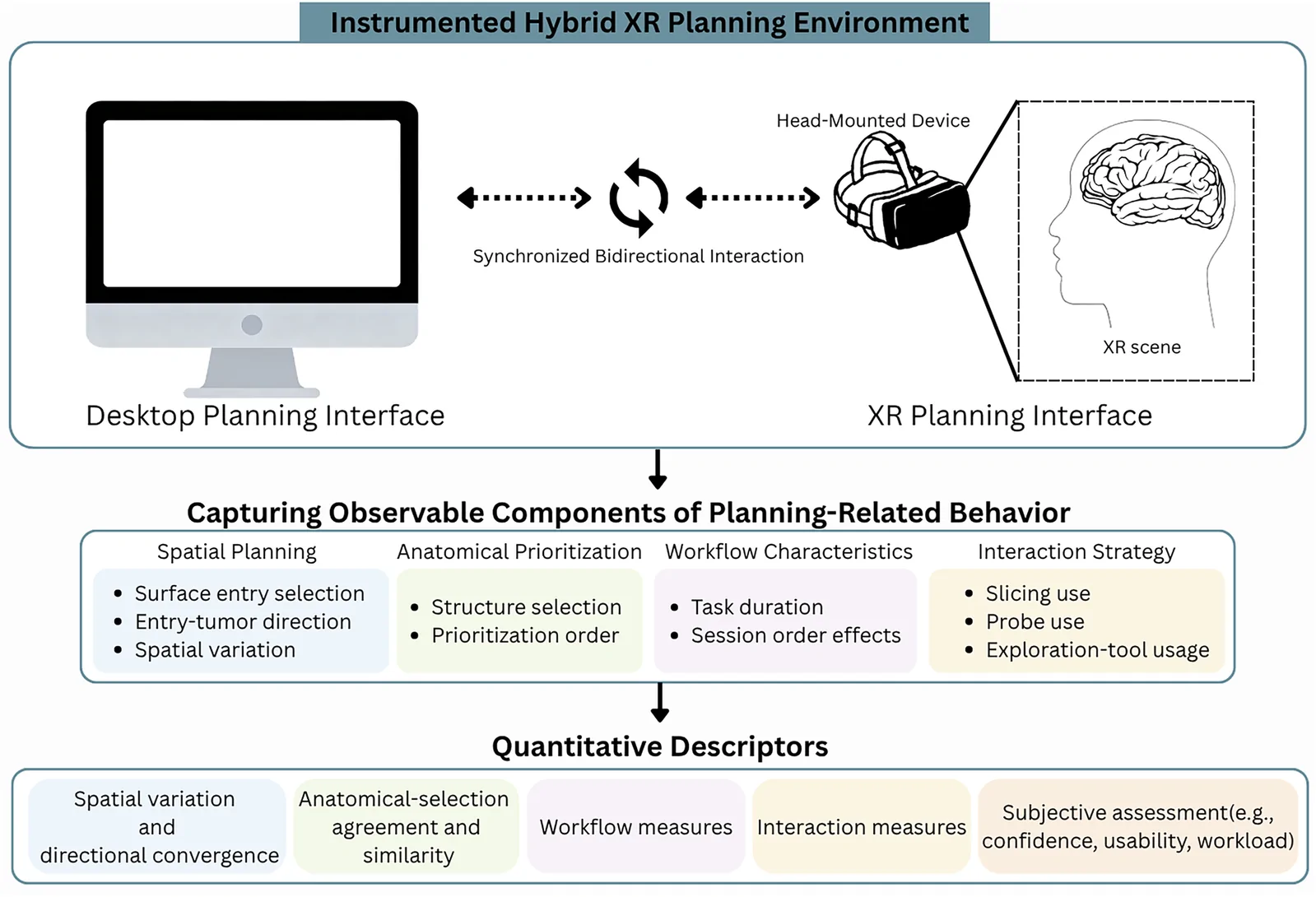
Conceptual overview of the instrumented hybrid desktop–XR planning environment and the observable planning-related measures captured across spatial planning, anatomical prioritization, workflow, interaction, and subjective-assessment domains.

The desktop workspace provided conventional two- and three-dimensional image review, anatomical visibility control, and planning-parameter adjustment. The XR workspace supported immersive inspection of patient-specific anatomical models, direct spatial manipulation, localized probe-based exploration, movable orthogonal cross-sectional planes, and surface entry-region planning. Participants could move between desktop-based review and immersive exploration during the planning workflow while maintaining a synchronized planning scene across both environments (Figure 2).

**Figure 2:**
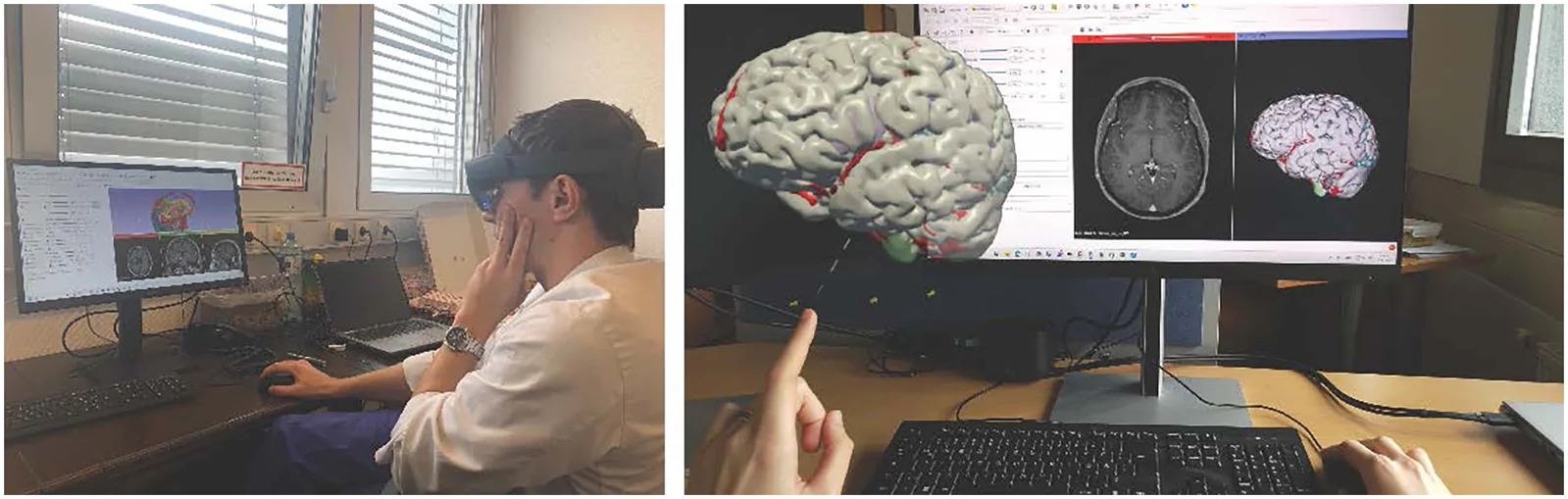
Evaluation setup and hybrid desktop–XR environment. **(A)** Participant setup with head-mounted display and synchronized desktop planning interface. **(B)** User viewpoint showing the desktop interface connected to the interactive three-dimensional anatomical model.

Three interaction tools supported planning-oriented spatial assessment. The probe-based tool enabled localized internal exploration by creating an inspection corridor from a user-defined spherical marker toward the center of the head model (Figure 3A). The cross-sectional tool enabled inspection of one orthogonal cutting plane at a time, with plane position synchronized between Unity and the corresponding slice position in 3D Slicer (Figure 3B). The entry-region planning tool enabled placement of a preferred surface entry marker and inspection of the resulting entry–tumor-center direction through stepwise model cutouts (Figure 3C).

**Figure 3:**
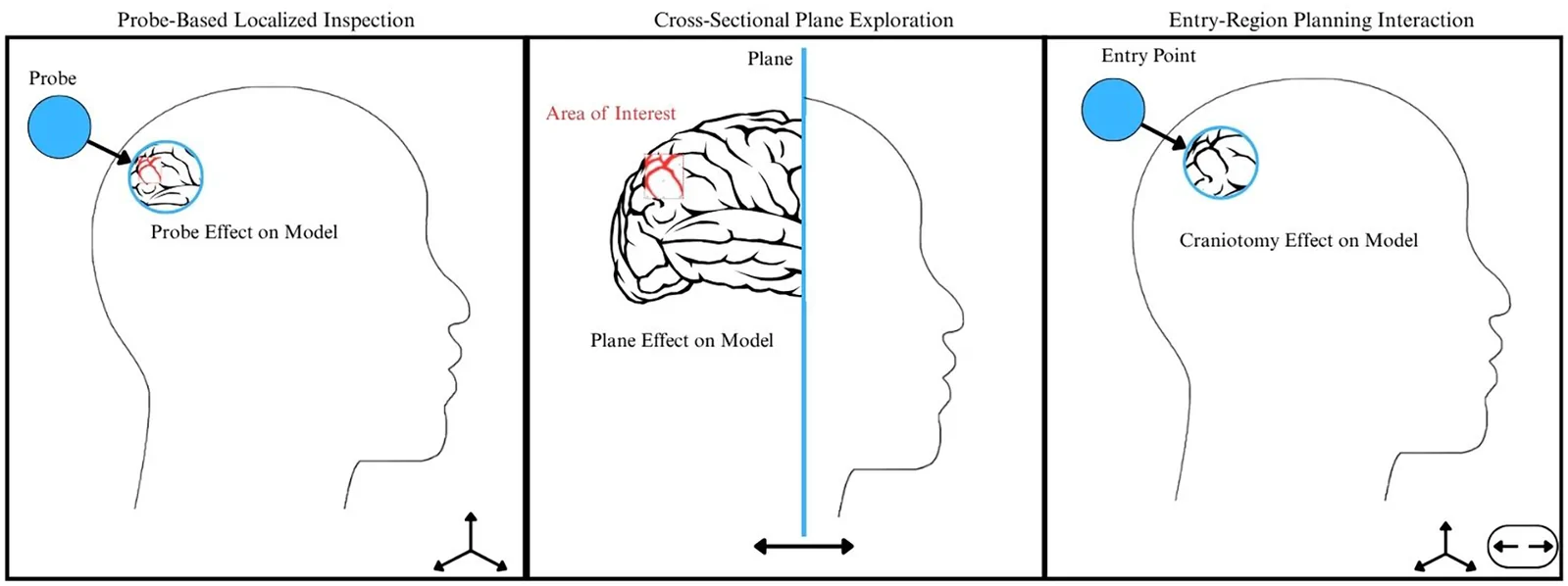
Conceptual overview of XR interaction modes. **(A)** Probe-based localized exploration, **(B)** synchronized orthogonal cross-sectional slicing, and **(C)** entry-region planning interaction using stepwise corridor-based cutouts toward the tumor center.

### 2.2 Patient data preparation

Two intra-axial brain tumor cases were included: one glioblastoma (GBM) and one low-grade glioma (LGG). The cases were selected by an experienced neurosurgeon to provide anatomically distinct planning scenarios involving lesions in relation to vascular and eloquent structures. They were intended as patient-specific evaluation scenarios rather than as a basis for comparing surgical-planning strategies between GBM and LGG. Both cases were retrospective surgical cases for which the clinical decision to proceed with surgery had already been made; lesions managed non-surgically were not included in the present evaluation. A separate non-study case was used for participant training.

Patient-specific planning scenes were prepared from preoperative MRI data, with CT data incorporated when available for skull reconstruction. Anatomical structures relevant to approach planning included tumor tissue, brain anatomy, vascular structures, skull and skin surface representations, dura, and selected eloquent regions. An additional infiltration-zone representation was available for the GBM case. Functional MRI and diffusion-based tractography were not incorporated into the standardized planning scenes; availability of the corresponding source data was not consistent across the two retrospective cases.

Segmentations were generated using manual and semi-automatic workflows in 3D Slicer, reviewed and confirmed by a neurosurgeon before evaluation, and converted into three-dimensional surface models for synchronized visualization and interaction across the desktop and XR environments (Figure 4).

**Figure 4:**
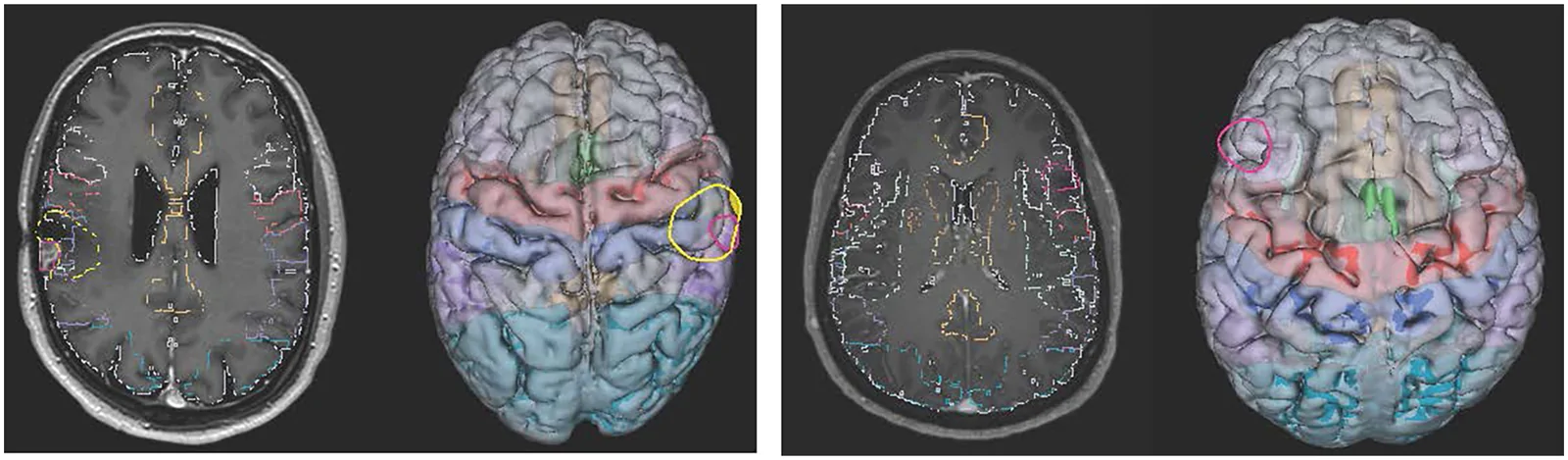
Prepared planning datasets used during evaluation. Axial MR images, segmentations, and three-dimensional planning models are shown for the GBM case **(A)** and LGG case **(B)**. Tumor tissue is shown in pink; the GBM case additionally includes the infiltration-zone representation in yellow.

### 2.3 Study design and procedure

This exploratory feasibility study evaluated whether participants could complete planning-oriented tasks within the synchronized desktop–XR framework and whether the framework could capture quantitative measures of observable planning behavior during use. Feasibility was primarily assessed by successful completion of both evaluation cases without session-ending technical interruption following standardized training. Additional exploratory outcomes included task duration, anatomical prioritization, entry–tumor-center direction, optional exploration-tool use, confidence, perceived risk, usability, and workload.

Twenty participants took part in the study: eight neurosurgeons, six neurosurgical residents, and six medical students. Participants were recruited across different levels of clinical experience to support exploratory comparison of observable planning-related behavior; medical students represented a lower-experience comparison group rather than being intended as routine clinical users of the framework. Eligibility required medical training or enrollment in medical studies and no prior exposure to the presented framework. Previous experience with XR or three-dimensional planning software was recorded but was not used as an exclusion criterion. Participant characteristics are summarized in Table 1.

**Table 1:** Participant demographics and prior experience characteristics

Characteristic	Category	*N* (%)
Professional role	Neurosurgeon	8 (40%)
	Neurosurgical resident	6 (30%)
	Medical student	6 (30%)
Age group (years)	18–24	4 (20%)
	25–34	12 (60%)
	35–44	4 (20%)
Gender	Male	13 (65%)
	Female	7 (35%)
Years of clinical experience	0	8 (40%)
	1–6	6 (30%)
	7–15	6 (30%)
Prior XR experience	Never used	8 (40%)
	Tried once or twice	9 (45%)
	Used several times	3 (15%)
Prior 3D planning experience	None/occasional	11 (55%)
	Regular user	9 (45%)
Visual correction	Yes (glasses, contact lenses)	9 (45%)
	No	11 (55%)

Each session began with standardized instruction and a familiarization phase using the separate training case. Participants were introduced to the synchronized desktop and XR environments and the available interaction tools before completing the two evaluation cases in counterbalanced order. During each case, participants could use the desktop and immersive XR views as part of the shared planning workflow. Participants were informed of the pathology of each case but were not provided with, and did not have access to, the original surgical plans; they therefore made their planning-related selections without reference to those plans.

Participants identified anatomical structures considered relevant for planning, ranked the selected structures by perceived priority, placed a preferred surface entry marker, explored the corresponding access direction, and rated confidence and perceived risk. Task start and end times were controlled using dedicated buttons in the Unity-based evaluation interface. After both evaluation cases, participants completed post-study questionnaires assessing spatial understanding, workflow coherence, interaction quality, usability, workload, and overall usefulness. The overall study sequence and per-case workflow are summarized in Figure 5.

**Figure 5:**
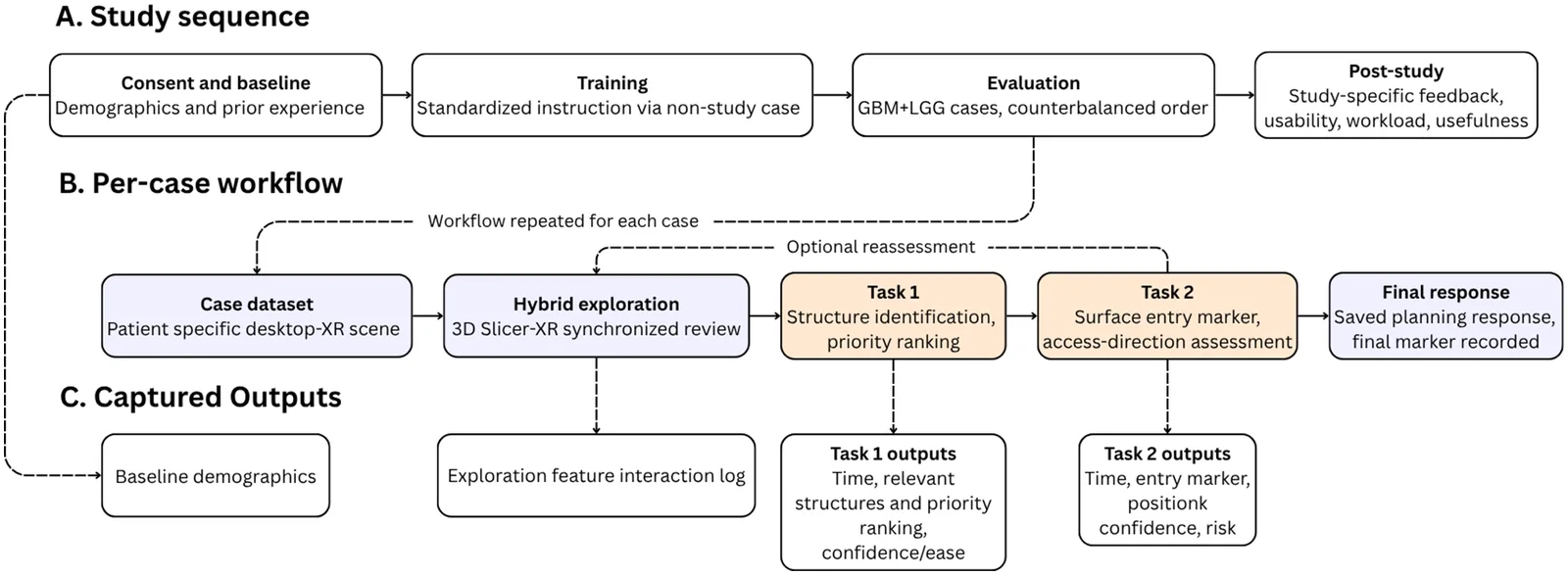
Study design and evaluation workflow. **(A)** Overall study sequence from baseline assessment and training to two counterbalanced evaluation cases and post-study questionnaires. **(B)** Repeated per-case workflow in the synchronized desktop–XR environment, including anatomical exploration, structure identification and priority ranking, surface entry-region placement, optional reassessment, and finalization. **(C)** Main captured outputs across baseline assessment, interaction logging, task-specific planning responses, and post-study evaluation.

### 2.4 Outcome measures

The evaluation dataset combined automatically logged workflow data, participant-entered planning responses, and post-study questionnaire data. Logged data included task timing, optional exploration-tool use, and surface entry-marker placement. Participant-entered responses included anatomical structure selection, priority ranking, confidence ratings, and perceived risk assessments entered through the Unity-based evaluation interface. Post-study questionnaires included the System Usability Scale (SUS) (37), an adapted NASA Task Load Index (NASA-TLX) (38), and additional study-specific questions. The adapted NASA-TLX assessed Mental Demand, Physical Demand, Temporal Demand, Performance, Effort, and Frustration using a 10-point response scale. The overall workload score was calculated as the arithmetic mean of the six dimension ratings.

The outcome measures were organized into five domains:

1. **Spatial planning**, based on participant-defined surface entry locations and derived entry–tumor-center directions;
2. **Anatomical prioritization**, based on selected structures and priority rankings;
3. **Workflow characteristics**, based on task duration;
4. **Interaction strategy**, based on optional exploration-tool use; and
5. **Subjective assessment**, including confidence, perceived risk, usability, workload, and study-specific ratings.

This organization was intended to capture complementary observable components of planning rather than reduce planning activity to a single outcome measure, and is summarized conceptually in Figure 1.

### 2.5 Data analysis and statistics

For trajectory analysis, the participant-defined surface entry marker and fixed tumor-model center were used to define an entry–tumor-center direction. For participant i, the normalized direction vector was calculated using Equation 1:

$$u_{i}=\frac{t-e_{i}}{\left\|t-e_{i}\right\|},$$ (1)

where t denotes the three-dimensional coordinates of the tumor center and $e_{i}$ the three-dimensional coordinates of participant i’s entry point.

Directional convergence C was calculated as the norm of the mean normalized vector across participants (Equation 2):

$$C=\left\|\frac{1}{N}\sum_{i=1}^{N}u_{i}\right\|,$$ (2)

where N is the number of participants. Values approaching 1 indicate stronger agreement in geometric access direction.

Directional dispersion D was defined using Equation 3:

D=1−C. (3)

Angular deviation was calculated as the angle between each participant-specific normalized vector and the case-specific mean direction. These metrics characterize geometric similarity of access directions rather than exact entry-point coincidence or clinical optimality.

Anatomical prioritization was summarized using selection frequencies and assessed using Fleiss’ κ (39). Agreement analyses were based on whether structures were selected as relevant rather than on agreement in rank order. Jaccard similarity and entropy were used as additional descriptive measures of selection consistency. Agreement categories followed Landis and Koch (40).

Because the study was exploratory and subgroup sizes were limited, analyses were considered hypothesis-generating. Task completion times, optional tool use, and subjective ratings were summarized descriptively. Within-participant comparisons used the Wilcoxon signed-rank test, and subgroup comparisons used the Kruskal–Wallis test where appropriate. For participant-level associations, repeated case-specific observations were averaged within participants before analysis using Spearman’s rank correlation coefficient. Matched-pairs rank-biserial correlation ($r_{rb}$) was reported as the effect-size measure for Wilcoxon signed-rank comparisons, while Spearman’s ρ served as the effect-size measure for rank correlations. For key estimates, 95% confidence intervals were obtained using participant-level percentile bootstrap resampling with 10,000 iterations. Statistical significance was defined as α=0.05.

### 2.6 Ethical approval

The study was approved by the institutional review board of the Medical University of Vienna (EK 409/2008). All procedures were conducted in accordance with the Declaration of Helsinki and institutional data-protection requirements. Written informed consent was obtained from all participants before participation.

## 3 Results

### 3.1 Framework operation and study completion

All 20 participants completed the training session and both evaluation cases without a session-ending technical interruption. No participant was excluded because of technical difficulties. Synchronization between the desktop and XR workspaces remained functional throughout the evaluation, enabling synchronized anatomical visualization, cross-sectional exploration, probe inspection, and entry-region planning within the same workflow.

The framework captured the predefined planning-related measures, including anatomical structure selection and prioritization, surface entry-point placement, task timing, optional exploration-tool use, and participant-reported confidence and perceived risk. Post-study questionnaires provided additional measures of usability, workload, and perceived usefulness.

A summary of the key behavioral, workflow, interaction, and subjective outcome measures is provided in Table 2; detailed findings are reported in the following sections.

**Table 2:** Summary of key behavioral and subjective outcome measures

Domain	Measure	Result
Framework feasibility	Task completion	20/20 participants completed training and both evaluation cases
Spatial planning	Directional convergence	GBM: 0.881 (95% CI: 0.686–0.987) LGG: 0.965 (95% CI: 0.935–0.990)
Spatial planning	Directional dispersion	GBM: 0.119 LGG: 0.035
Spatial planning	Median entry–tumor distance	GBM: 97.6 mm LGG: 146.4 mm
Anatomical prioritization	Fleiss’ κ	Overall: 0.397 GBM: 0.311 LGG: 0.408
Anatomical prioritization	Role-specific Fleiss’ κ	Neurosurgeons: 0.470 Residents: 0.425 Medical students: 0.345
Workflow	Structure-identification time	336.2 s to 219.5 s, p=0.000134, $r_{rb}=0.886$
Workflow	Entry-region planning time	90.0 s to 76.2 s, p=0.024, $r_{rb}=0.571$
Interaction	Optional exploration tools	Slicing: 6/20 participants Probe: 5/20 participants
Subjective evaluation	Spatial relationship interpretation	5.58 ± 1.43/7
Subjective evaluation	Access-direction confidence	5.28 ± 1.38/7
Subjective evaluation	SUS	Median 60.0, IQR 12.5
Subjective evaluation	Adapted NASA-TLX	Mean 3.46 ± 0.76/10

### 3.2 Trajectory planning behavior

Participant-defined surface entry locations varied within each case, while entry–tumor-center directions showed substantial convergence (Figure 6). The corresponding case-specific tumor and entry-point visualizations are shown in Figure 7. Directional convergence was higher descriptively in the LGG case, with a mean vector norm of 0.965 (95% bootstrap CI: 0.935–0.990) and directional dispersion of 0.035, compared with 0.881 (95% bootstrap CI: 0.686–0.987) and 0.119, respectively, for the GBM case.

**Figure 6:**
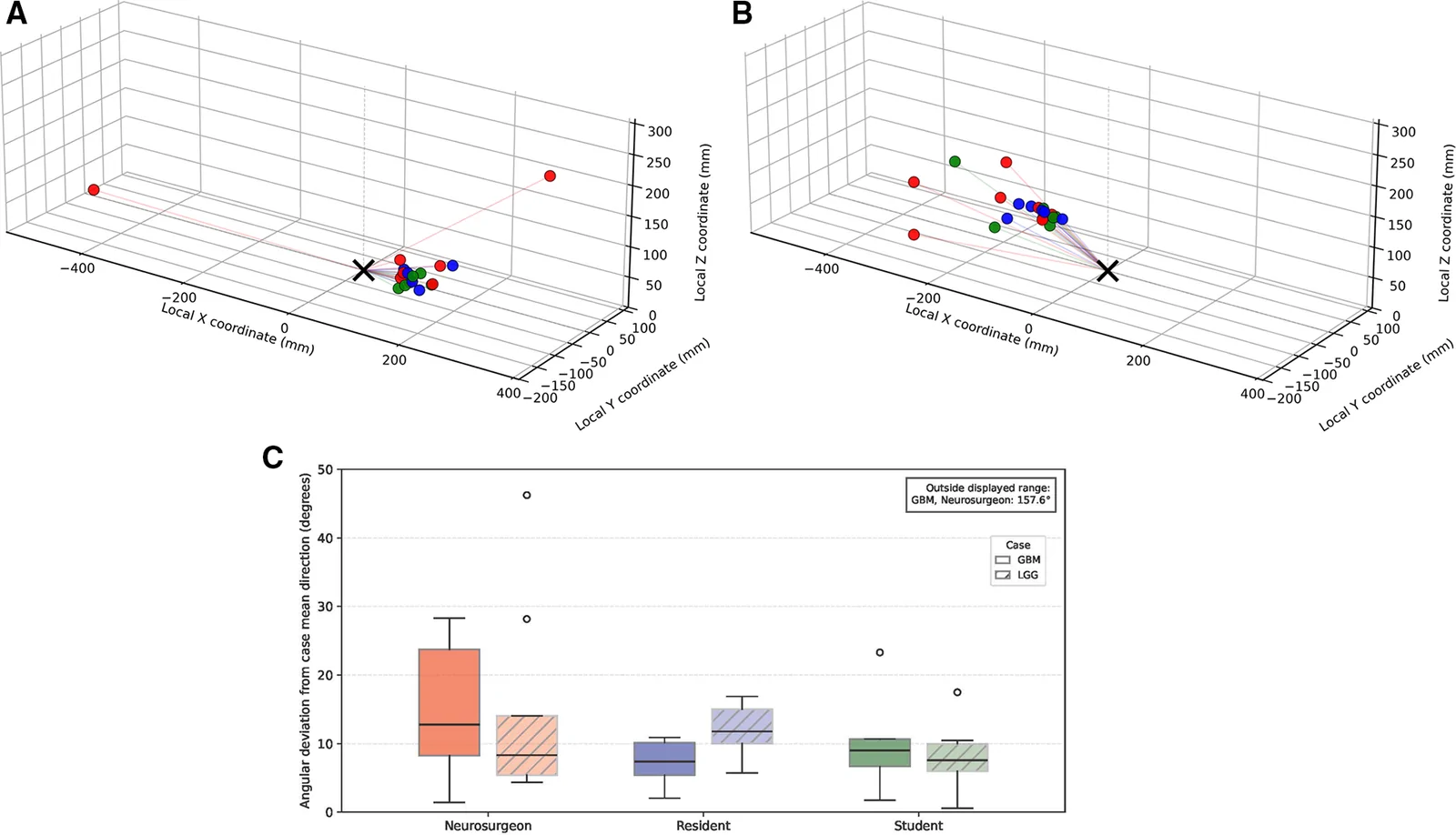
Directional variability and spatial distribution of selected entry points. **(A, B)** Three-dimensional entry-point distributions and corresponding entry–tumor-center directions for the GBM case **(A)** and LGG case **(B)**. Colors indicate participant role: red = neurosurgeon, blue = neurosurgical resident, and green = medical student. **(C)** Angular deviation from the case-specific mean direction by role and case. The y-axis was limited for readability; one GBM neurosurgeon value outside the displayed range is indicated in the annotation box and was retained in the analysis.

**Figure 7:**
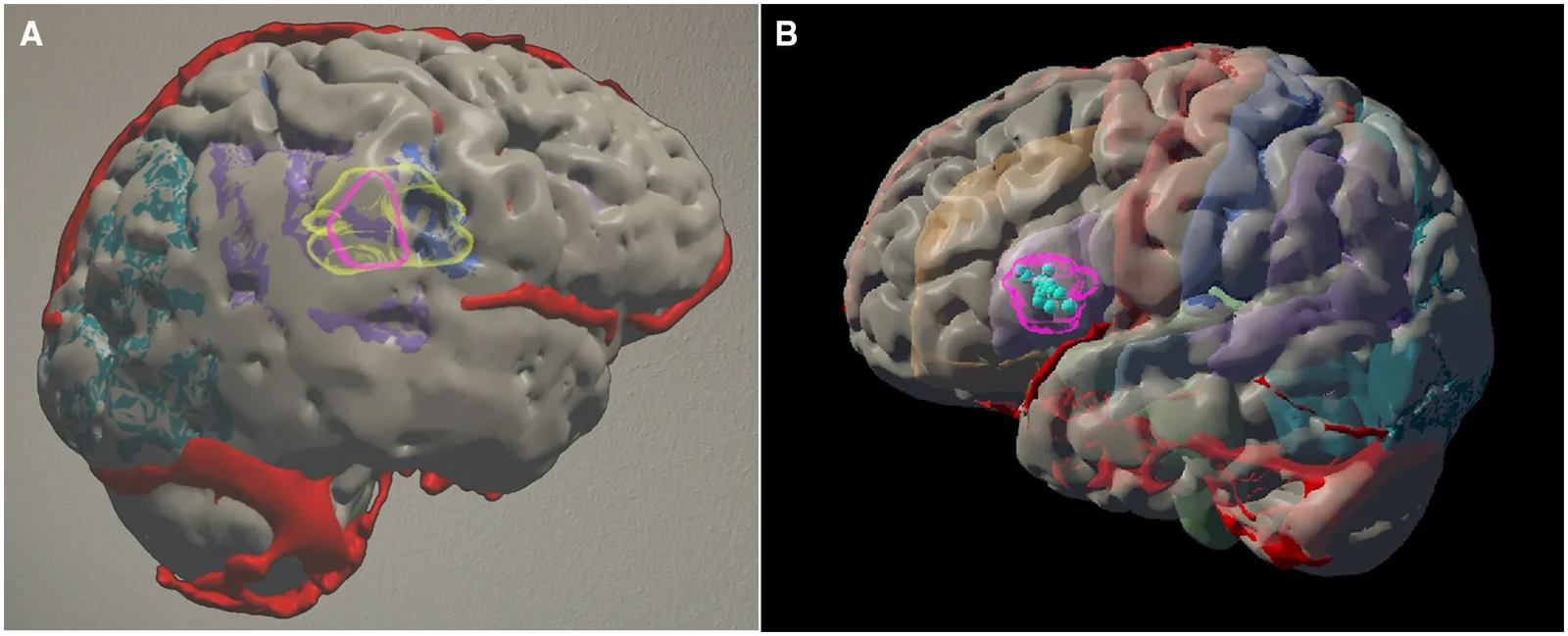
Tumor extent and anatomical entry-point visualization. **(A)** GBM case showing tumor core in pink and infiltration-zone representation in yellow. **(B)** LGG case showing semi-transparent brain tissue, segmented planning-relevant structures, tumor in pink, and participant-defined cortical entry points in blue.

Angular-deviation analysis showed the same case-dependent pattern, with lower mean angular deviation in the LGG case than in the GBM case (11.8${}^{\circ }$ vs. 17.6${}^{\circ }$). Median angular deviations were relatively similar (9.1${}^{\circ }$ vs. 10.0${}^{\circ }$), although the GBM case included one large outlying value of 157.6${}^{\circ }$.

Entry–tumor distances also differed between cases, with median distances of 97.6 mm for GBM and 146.4 mm for LGG. Exploratory comparisons between professional groups did not reveal clear systematic differences in trajectory direction or entry-point distribution, although subgroup sample sizes were limited.

Overall, surface entry locations varied between participants, whereas entry–tumor-center directions were more consistent, with higher directional convergence in the LGG case.

### 3.3 Anatomical structure prioritization and planning agreement

Across both evaluated cases, participants identified a mean of 3.45 ± 1.62 anatomical structures per session (median = 3.5, IQR = 2.25; range 0–7). The most frequently selected structures were blood vessels (77.5%), motor cortex (60.0%), Broca’s area (55.0%), and primary somatosensory cortex (45.0%) (Figure 8). Broca’s area was selected by all participants in the LGG case, while it was selected only occasionally in the GBM case. Priority rankings provided complementary information, with Broca’s area most frequently ranked as the highest-priority structure, followed by the motor cortex, Wernicke’s area, and primary somatosensory cortex.

**Figure 8:**
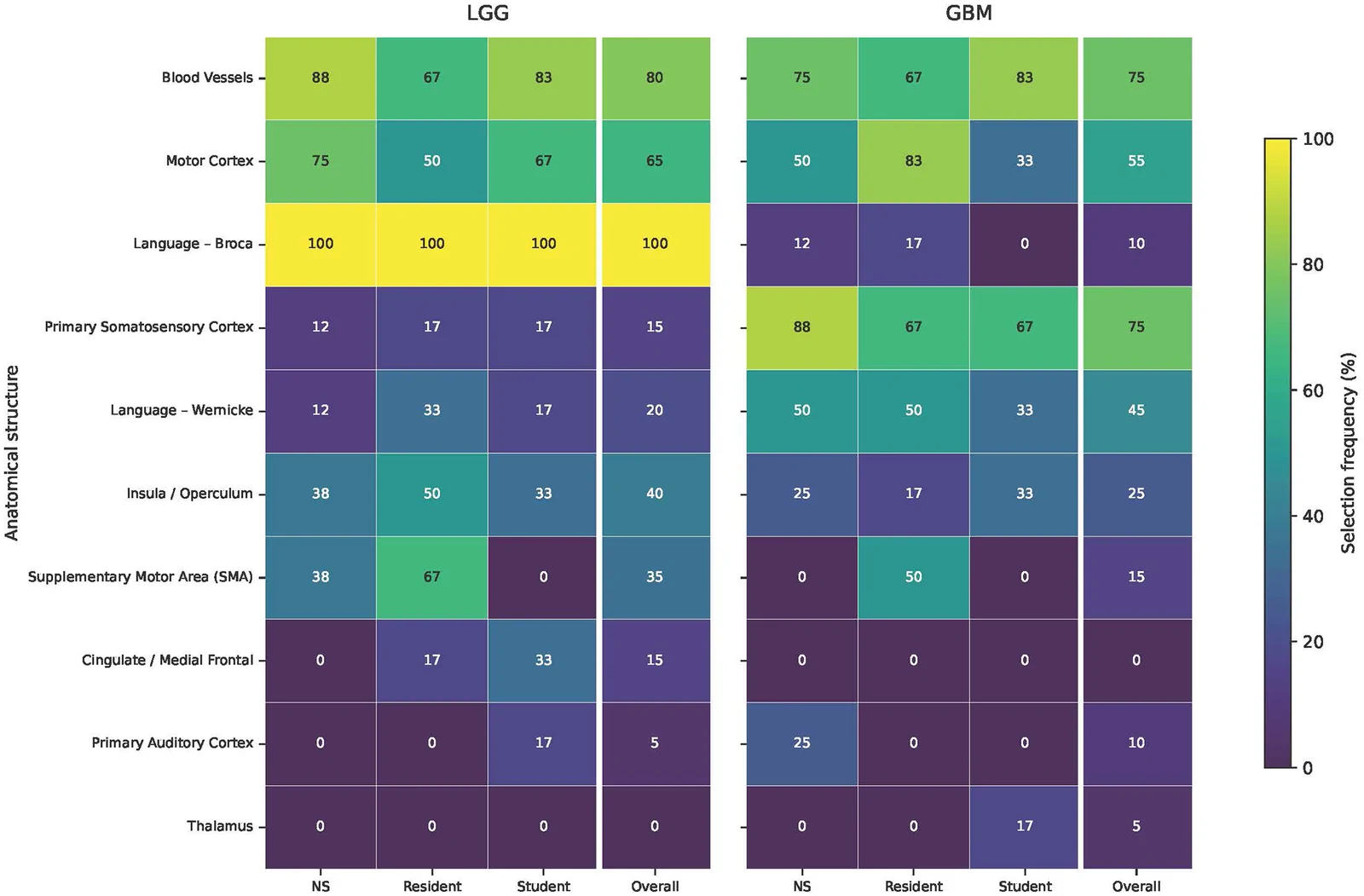
Anatomical structures identified as planning-relevant during Task 1. Heatmaps show selection frequencies by participant role and case, with the overall column summarizing selections across all roles. Values indicate the percentage of sessions in which each structure was selected.

Structure-selection patterns differed between cases. In the LGG case, participants more frequently selected language-related regions, with a mean of 3.75 selected structures per session. The GBM case showed more frequent selection of vascular and sensorimotor anatomy, with a mean of 3.15 structures per session.

Overall agreement in whether anatomical structures were considered relevant was fair, with Fleiss’ κ=0.397 across both cases. Case-specific agreement ranged from fair in the GBM case (κ=0.311) to moderate in the LGG case (κ=0.408). By participant role, κ was 0.470 among neurosurgeons, 0.425 among residents, and 0.345 among medical students.

Additional Jaccard-similarity and entropy analyses showed the same descriptive pattern. Mean pairwise Jaccard similarity was 0.523 for medical students, 0.595 for residents, and 0.611 for neurosurgeons, while entropy was 0.644, 0.562, and 0.535, respectively.

Together, these descriptive measures showed variation in anatomical structure-selection consistency across participant groups. However, these differences should not be interpreted as evidence of expertise-related effects.

### 3.4 Workflow and interaction behavior

Task completion times decreased between the first and second evaluation case (Figure 9A). For anatomical structure identification, median completion time decreased from 336.2 s (IQR = 238.8) to 219.5 s (IQR = 105.9; Wilcoxon signed-rank test, W=12.0, p=0.000134, matched-pairs rank-biserial $r_{rb}=0.886$). The median within-participant reduction was 141.2 s (95% bootstrap CI: 84.5–213.0 s). For entry-region planning, median completion time decreased from 90.0 s (IQR = 77.5) to 76.2 s (IQR = 44.0; W=45.0, p=0.024, matched-pairs rank-biserial $r_{rb}=0.571$). The median within-participant reduction was 24.7 s (95% bootstrap CI: −2.8–50.5 s). These findings indicate a significant session-order effect, consistent with increasing familiarity with the workflow. No significant task-duration differences were observed between the GBM and LGG cases for anatomical structure identification (p=0.756) or entry-region planning (p=0.097).

**Figure 9:**
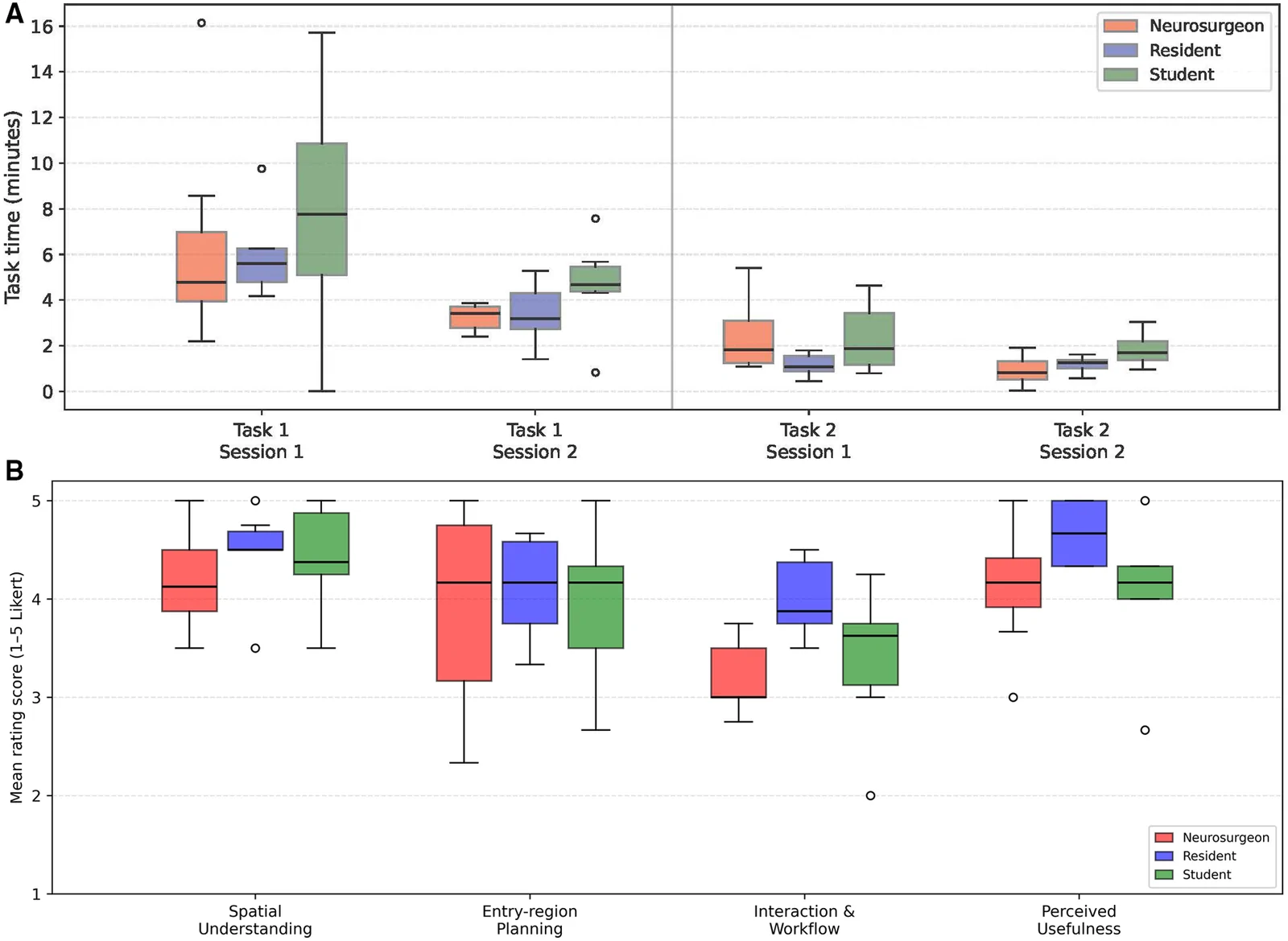
Workflow performance and planning-related user assessments. **(A)** Comparison of task completion times between Session 1 and Session 2 for the structure identification and entry-region planning tasks. **(B)** Questionnaire category scores reflecting perceived spatial support, planning assistance, interaction quality, and overall usefulness of the framework, shown by participant role.

Descriptively, medical students required longer completion times than residents and neurosurgeons. For anatomical structure identification, mean task time was 375 s for medical students, 286 s for neurosurgeons, and 282 s for residents. For entry-region planning, mean task time was 126 s, 100 s, and 70 s, respectively. These subgroup patterns should be interpreted descriptively because of the limited sample size.

Optional exploration tools were used selectively. Six of 20 participants (30%) used the slicing mode, with a median interaction time of 279.8 s among users. Five participants (25%) used the probe mode, with a median interaction time of 79.6 s among users. Because these tools were optional and self-selected, their use was analyzed descriptively.

### 3.5 Subjective evaluation

Participants reported generally positive assessments of spatial support (Figure 9B). Spatial relationship interpretation received a mean rating of 5.58 ± 1.43 on the seven-point scale, while access-direction confidence was 5.28 ± 1.38.

Neurological-risk ratings differed significantly between cases (Wilcoxon signed-rank test, W=18.0, p=0.027, matched-pairs rank-biserial $r_{rb}=0.657$). Because lower values indicated higher perceived neurological risk on this scale, the LGG case was rated as neurologically riskier than the GBM case. Overall/procedural risk and access-direction confidence did not differ significantly between cases.

Confidence in anatomical interpretation and confidence in access-direction planning were positively associated at the participant level (Spearman ρ=0.685, p=0.000869, 95% bootstrap CI: 0.355–0.869).

The median SUS score was 60.0 (IQR = 12.5), indicating room for usability and interaction refinement. The adapted NASA-TLX indicated low-to-moderate reported workload, with a mean score of 3.46 ± 0.76 on the 10-point scale. Medical students showed descriptively higher mental demand and effort than neurosurgeons and residents, whereas physical demand was similar across groups. Given the small subgroup sizes, these role-related differences were interpreted descriptively.

Study-specific feature ratings were generally positive, ranging from 4.14 to 4.83 on the five-point scale. Exploratory role comparisons did not identify statistically significant differences for individual features (Kruskal–Wallis tests, all p>0.05).

## 4 Discussion

### 4.1 Principal findings

This study evaluated a synchronized desktop–XR framework for patient-specific preoperative neurosurgical planning and demonstrated the feasibility of capturing multiple observable components of planning activity within a shared workflow. Across 20 participants, the framework captured spatial access-direction choices, anatomical structure selection, workflow timing, optional interaction-tool use, and subjective assessments. The findings therefore extend beyond assessment of XR visualization or usability alone by demonstrating how an XR-supported planning environment can also be instrumented to record planning-related behavior.

An important finding was that different measures captured different aspects of planning. Surface entry locations varied between participants, whereas entry–tumor-center directions showed substantial convergence. This suggests that direction-based measures can capture agreement that would not be apparent from entry-point location alone and that evaluating only the selected surface entry point may underestimate similarities between planning strategies. Conversely, directional convergence does not establish that the convergent direction was clinically preferable, because no reference trajectory or independent criterion of optimality was available.

Anatomical structure selection provided a complementary behavioral dimension, revealing both commonly prioritized structures and variation across participants.

The Jaccard and entropy measures provided additional descriptions of variation across participant groups. However, the exploratory cohort does not permit these differences to be attributed specifically to clinical expertise.

These findings highlight the multidimensional nature of preoperative planning, in which patient-specific anatomy, potential access routes, and structures relevant to the proposed approach contribute to planning-related decisions rather than a single observable endpoint. Previous work on surgical process modeling and inter-surgeon variability has demonstrated that procedural activities and spatial selections can vary between practitioners and can be represented through structured behavioral data (28–31). Surgical data science and computer-assisted intervention research have further emphasized the value of systematically capturing procedural and contextual information to characterize variation and support development of computer-assisted approaches (32, 33).

### 4.2 Case-dependent and experience-related variation

The differences between the two cases further demonstrate why multidimensional measurement may be useful. Directional convergence and anatomical-selection agreement were both higher in the LGG case than in the GBM case. The LGG case was characterized by more frequent selection of language-related anatomy, whereas the GBM case showed greater emphasis on vascular and sensorimotor structures.

These differences should be interpreted as case-specific observations rather than general differences between glioma types, because only one case of each type was evaluated. The study was not designed to compare surgical-planning strategies between GBM and LGG, for which tumor biology, location, functional anatomy, and other clinical factors may lead to different surgical considerations. Nevertheless, they illustrate that the observable planning measures captured differences associated with the anatomical configuration of the presented cases. This is relevant to the proposed framework because an instrumented planning environment should preserve variation arising from the planning context rather than reduce user behavior to a single performance measure.

The greater anatomical-selection consistency observed among neurosurgeons is also potentially relevant to the study’s objective of characterizing variation across levels of clinical experience. However, the present data do not establish a causal relationship between clinical experience and any individual behavioral measure. Participants also differed in prior XR and three-dimensional planning experience, and the cohort was not sufficiently large to disentangle these factors. The experience-related findings should therefore be regarded as observable group patterns rather than evidence that the framework provides a validated measure of expertise.

### 4.3 Workflow and interaction behavior

Workflow measures provided an additional dimension of observable planning behavior that is not readily available from a static visualization assessment. The reduction in task duration between sessions is consistent with increasing familiarity with the experimental environment, although it does not establish improved planning efficiency or performance. Optional interaction-tool use was heterogeneous, indicating that participants adopted different strategies for exploring patient-specific anatomy. Because these tools were optional and self-selected, their use cannot establish that a particular interaction modality improved planning or decision quality. Nevertheless, the ability to record such interactions demonstrates how the instrumented environment can capture differences in users’ exploration strategies during planning.

This capability complements previous work in surgical process modeling and surgical data science, where structured representations of activities and interactions have been used to characterize procedural variation and support the development of computer-assisted systems (28, 31–33). In the present setting, this principle is extended to the preoperative planning phase, where relevant activities occur before the surgical procedure itself.

### 4.4 Subjective experience and usability

Subjective measures provided complementary information about participants’ experience of the hybrid planning environment. Ratings indicated generally favorable perceptions of spatial relationship interpretation and access-direction confidence, while the adapted NASA-TLX indicated relatively low-to-moderate reported workload. At the same time, the median SUS score of 60.0 indicates room for further usability and interaction refinement, and the current interface should therefore not be considered fully optimized for clinical planning.

These findings support the feasibility of the environment for planning-oriented spatial assessment but do not establish clinical benefit. In particular, subjective confidence or perceived spatial understanding cannot demonstrate that participants made more accurate or clinically preferable decisions. Their principal value in the present study is therefore contextual: they characterize how participants experienced the instrumented workflow alongside the objectively captured behavioral measures.

### 4.5 Relationship to previous XR research

Previous neurosurgical XR studies have focused on three-dimensional visualization, spatial understanding, education and training, preoperative planning, navigation, usability, and technical feasibility (9, 14–19, 21, 22, 26, 27). Mixed-reality systems have demonstrated the feasibility of patient-specific visualization, presurgical mapping and navigation, and integration with established platforms such as 3D Slicer and Microsoft HoloLens 2 (21, 22, 26).

The present work addresses a complementary question: whether an XR-supported planning environment can be instrumented to capture observable components of planning activity. By recording spatial, anatomical, workflow, and interaction-related measures alongside subjective assessments, the framework extends XR evaluation beyond visualization and usability toward quantitative characterization of planning-related behavior.

This approach is consistent with surgical process modeling and studies of inter-surgeon variability, which have demonstrated the value of structured behavioral data for characterizing procedural variation (28–31). Related work in computer-assisted intervention and surgical data science further supports systematic capture of procedural and contextual information (32, 33). Here, this perspective is applied to the preoperative planning phase without assuming that the resulting measures represent planning quality or clinical expertise.

### 4.6 Relevance to XR integration and healthcare deployment

A broader challenge for XR in healthcare is not only demonstrating useful immersive visualization, but also integrating it into established clinical workflows. The present framework addresses this challenge through an integration-oriented architecture that combines conventional desktop-based image review with synchronized immersive exploration rather than replacing the established planning environment. An additional practical characteristic of the demonstrated configuration is that XR interaction was performed while participants remained seated at the conventional planning workstation. Thus, the immersive component did not require a dedicated room-scale installation or additional physical workspace beyond the existing desktop planning environment. This configuration may therefore support incremental incorporation of XR into existing workflows. Previous work has demonstrated real-time integration between 3D Slicer and HoloLens 2 (26), while broader XR research has increasingly considered the transition from isolated demonstrations toward healthcare integration (24, 25). The contribution here is therefore not a fully XR-native clinical pipeline, but a framework in which heterogeneous components can participate in a synchronized planning workflow while retaining their respective functions. The instrumentation layer adds a further deployment-relevant capability. By recording spatial, anatomical, workflow, and interaction-related information, the framework converts selected aspects of an experiential XR workflow into structured data. This may support future evaluation of XR-supported planning, comparison of interaction strategies, and investigation of whether observable behaviors correspond to clinically meaningful constructs.

The present study does not, however, demonstrate routine clinical deployment, multidisciplinary workflow integration, institutional implementation, long-term adoption, or clinical benefit. It therefore establishes the feasibility and methodological relevance of an integration-oriented XR framework, rather than a clinically deployable solution. Evaluation in realistic clinical settings will be required to determine whether immersive visualization can be incorporated without unacceptable workflow burden and whether the captured behavioral measures provide practical value to clinical teams.

### 4.7 Limitations and future validation

Several limitations constrain interpretation. The exploratory cohort was small, with limited experience-group sizes, and participants also differed in prior XR and three-dimensional planning experience. Only two patient-specific cases were evaluated, limiting generalizability across anatomical configurations, neurosurgical pathologies, hemispheric and functional contexts, and surgical scenarios. The standardized planning scenarios also did not incorporate all additional imaging modalities that may contribute to tumor characterization and surgical planning, such as magnetic resonance spectroscopy, functional MRI, or diffusion-based tractography. Future clinically focused evaluations could investigate the integration of such complementary imaging information where clinically indicated.

The study also lacked a desktop-only control condition; therefore, it cannot establish whether XR improves planning performance relative to conventional visualization. Furthermore, the behavioral measures were not validated against expert reference plans, predefined clinical criteria, intraoperative observations, or patient outcomes. Consequently, measures such as directional convergence, anatomical-selection consistency, and task duration should be interpreted as observable planning descriptors rather than indicators of planning quality or expertise.

The evaluation investigated short-term use in a controlled experimental setting and therefore does not establish how the synchronized desktop–XR workflow would integrate into routine multidisciplinary clinical planning. In addition, the reduction in task duration between evaluation sessions may reflect familiarization with the experimental environment rather than improved planning performance.

Future studies should evaluate larger and more diverse clinical cohorts and patient-specific cases, including greater representation of practicing neurosurgeons and neurosurgical residents involved in neuro-oncological surgery and broader recruitment across clinical centres with substantial brain-tumor caseloads where feasible, compare hybrid and conventional planning workflows, and investigate construct validity against expert reference plans and clinically grounded criteria.

## 5 Conclusion

This study demonstrated the feasibility of an instrumented hybrid desktop–XR framework that integrates conventional desktop-based image review with immersive exploration of patient-specific neurosurgical anatomy. By synchronizing planning states and capturing spatial, anatomical, workflow, interaction, and subjective measures, the framework makes selected otherwise implicit components of preoperative planning observable and quantifiable.

The findings support the use of such instrumentation to characterize variation in planning-related behavior across participants and patient-specific cases. However, the recorded measures should currently be regarded as descriptive behavioral measures rather than validated indicators of surgical expertise, planning quality, or clinical correctness.

The framework therefore provides a methodological basis for subsequent studies involving larger cohorts, more diverse clinical cases, conventional-workflow comparisons, and validation against expert or clinical criteria. Such work may clarify whether observable planning-related behaviors captured through XR can provide clinically meaningful information about preoperative planning.

## Data Availability

The datasets presented in this article are not readily available because of the ethical, privacy, and institutional data-protection restrictions. Patient-specific imaging data are subject to the applicable ethical approval and institutional data-governance requirements. Requests to access the datasets should be directed to Andreas Daniel Hartl (A.Hartl@fh-kaernten.at), corresponding author.
